# Sensory Circumventricular Organ Insulin Signaling in Cardiovascular and Metabolic Regulation

**DOI:** 10.3390/cimb47080595

**Published:** 2025-07-29

**Authors:** Han Rae Kim, Jin Kwon Jeong, Colin N. Young

**Affiliations:** Department of Pharmacology and Physiology, George Washington University School of Medicine and Health Sciences, Washington, DC 20037, USA; jinkwon0911@gmail.com (J.K.J.); colinyoung@gwu.edu (C.N.Y.)

**Keywords:** CNS insulin receptor, sensory circumventricular organs, blood-brain barrier, metabolism, cardiovascular regulation

## Abstract

Central nervous system (CNS) insulin signaling is involved in a broad array of cardiometabolic physiology, including glucose and lipid metabolism, feeding, energy expenditure, and blood pressure regulation. A key role for hypothalamic neuroendocrine and autonomic centers in regulating insulin-associated cardiovascular and metabolic physiology has been highlighted. However, it is still unclear which CNS site(s) initiate insulin-dependent neural cascades. While some investigations have suggested that circulating insulin can access hypothalamic regions by crossing the blood-brain barrier, other studies point to a necessity of other brain areas upstream of the hypothalamus to initiate central insulin actions. In this context, accumulating evidence points to a possible involvement of the sensory circumventricular organs (CVOs), unique areas located outside of the blood-brain barrier, in insulin-dependent cardiometabolic homeostasis. Here, the multifaceted roles for the sensory CVOs in cardiovascular and metabolic regulation, with a special emphasis on insulin receptor pathways, are discussed.

## 1. Introduction

Metabolic syndrome is a worldwide health concern encompassing complex metabolic and cardiovascular problems, including obesity, diabetes, and hypertension [1]. The underlying causes are multifaceted; however, alterations within the central nervous system (CNS) are directly implicated in metabolic syndrome [2,3]. In particular, CNS signaling by the hormone insulin, which is well known for its role in regulating glucose homeostasis and metabolism [4,5], plays a central role not only in basal cardiometabolic regulation, but is emerging as a culprit in metabolic syndrome progression [6,7]. However, our understanding of CNS insulin action remains unclear. Indeed, the influences of insulin on the brain are complex. For instance, studies in humans demonstrated that insulin does not directly affect glucose transport across the blood-brain barrier (BBB) or cerebral glucose metabolism [8]. Conversely, basal insulin levels have been revealed to be crucial for brain glucose uptake in humans, highlighting the constant and essential role of insulin in maintaining brain function [9]. Therefore, it is crucial to consider insulin’s multifaceted effects on the brain. This includes understanding potential roles for insulin within the CNS, beyond the canonical cellular metabolic regulation, that may influence peripheral physiological processes beyond the brain.

Appropriate information processing in the CNS is achieved through multiple layers of cellular and regional networks, and most efforts have focused on hypothalamic neuroendocrine and autonomic nuclei as integration sites for insulin-related cardiometabolic control. However, the brain site(s) that sense and initiate insulin-dependent signaling remain understudied. Several investigations have suggested that insulin penetrates the BBB, and therefore, it can travel directly from the circulation to target brain regions [10]. Nevertheless, other investigations have also indicated that deep brain regions including the hypothalamic paraventricular nucleus (PVN), a regulatory site for neuroendocrine and autonomic output, is not a direct target for insulin [11]. These investigations also pointed to a possible involvement of insulin-associated action in brain regions upstream of the PVN. In this regard, the hypothalamic arcuate nucleus (ARC) has been highlighted to sense and initiate insulin-mediated cardiometabolic regulation [12]. Importantly, the ARC is fully protected by the BBB [13], although several reports have indicated that circulating cardiometabolic factors can access the ARC via the median eminence (ME) [14,15]. While a role for the arcuate cannot be excluded, emerging evidence further points to the sensory circumventricular organs (CVOs) as potential key CNS sites through which insulin signaling influences cardiometabolic regulation. Thus, the focus of this review is to highlight existing literature specifically related to insulin action in the sensory CVOs that may allow for the inference and/or explanation of their involvement in cardiovascular and metabolic regulation.

## 2. Sensory CVOs as a Potential Key Site for Insulin Action

**Figure 1:**
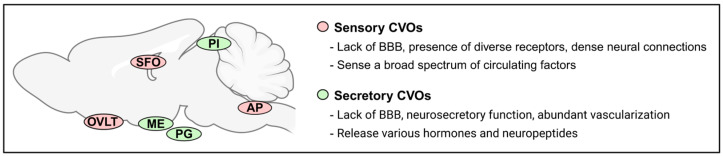
Schematic illustrating the anatomical location of the sensory and secretory circumventricular organs in the midsagittal section of the mouse brain. SFO, subfornical organ; OVLT, organum vasculosum lamina terminalis; AP, area postrema; PI, pineal gland; ME, median eminence; PG, pituitary gland; BBB, blood-brain barrier. Image was created with Biorender.com; accessed on 25 July 2025.

Specialized endothelial cells in the CNS microvasculature are surrounded by dense and continuous tight junctions and establish a non-fenestrated barrier between the bloodstream and CNS [16]. This BBB with meager vesicular transport capacity plays a critical role in protecting the brain from circulating toxins and pathogens. The BBB is established in all CNS structures except for the CVOs; a group of tiny brain structures including the ME, pineal gland, pituitary gland, area postrema (AP), organum vasculosum lamina terminalis (OVLT), and subfornical organ (SFO) [17,18]. The CVOs are classified into sensory types, which sense various circulating factors, and secretory types, which release various hormones and neuropeptides (Figure 1) [18]. Among the CVOs, the AP, OVLT and SFO are known as the sensory CVOs as they have the cellular machinery to sense a broad spectrum of circulating factors from the bloodstream [18]. Relative to the rest of the CNS, the capillary system in the sensory CVOs is more permeable. Loose tight junctions and fenestrated capillaries are present; therefore, peripheral molecules can directly access the CVOs from the circulation [19]. Importantly, the sensory CVOs establish complex neural networks with other brain regions implicated in neuroendocrine and autonomic homeostasis and play key roles in regulating thirst, drinking behavior, metabolic, and cardiovascular physiology [20].

Brain insulin signaling, via insulin receptors, is broadly implicated in cardiovascular and metabolic physiology. For example, numerous investigations have indicated that central insulin reduces feeding behavior, body weight gain, and hepatic glucose production [6,21]. At the same time, brain insulin also activates the sympathetic nervous system to increase blood pressure, modulate baroreflex control, and elevate heart rate [6,22,23]. Insulin receptors are widely, but not ubiquitously, distributed throughout the CNS with brain region-dependent differential expression patterns [24,25]. Importantly, visualization of CNS insulin binding sites using radioactively labeled insulin indicated that all sensory CVOs are a direct target for circulating insulin [26,27,28,29].

These findings have been further validated with recent research approaches including electrophysiology [30], histology [6,31], region-specific transcriptome and gene expression analysis [32,33], and manipulation of insulin receptors with pharmacological and/or genetic targeting [6,34]. Thus, a growing body of evidence points to the sensory CVOs as anatomically situated to detect circulating insulin, and as such, regulate cardiometabolic physiology.

## 3. Sensory CVOs Insulin Receptors in Cardiovascular Regulation

### 3.1. SFO

Located along the midline of the brain adjacent to the lateral ventricle, the SFO is extensively recognized for its ability to regulate cardiovascular function, body fluid homeostasis, and thirst behavior [35]. Moreover, due to the complex SFO molecular network that senses and integrates circulating signals, alterations in the SFO are implicated in the development of cardiovascular diseases encompassing several neurogenic forms of hypertension, including angiotensin-II (Ang-II) [36] and deoxycorticosterone acetate-salt-dependent hypertension [37], as well as heart failure [38]. For example, in the context of hypertension development, a current consensus is that the SFO provides excitatory inputs to downstream hypothalamic and brainstem nuclei to influence neuroendocrine and autonomic outputs [35] and subsequently elevate blood pressure, although the precise cellular mechanisms within the SFO as well downstream nuclei in neurogenic hypertension development remains unclear.

Given the strong links between insulin, sympathetic outflow, and blood pressure regulation, recent experiments examined the influence of SFO insulin receptors on cardiovascular regulation [6]. Removal of SFO insulin receptors in young healthy mice resulted in a deleterious metabolic state including increased weight gain and adiposity, hepatic steatosis, and hypertriglyceridemia [6]. Despite this, SFO insulin receptor ablation was associated with a modest, yet sustained reduction in arterial blood pressure without affecting heart rate. This hypotensive action appeared to be time-of-day-dependent, with the greatest lowering of blood pressure evident during the dark phase [6]. These results suggest that tonic insulin action within the SFO is necessary for the maintenance of arterial blood pressure. Moreover, these findings are intriguing considering that hyperinsulinemia has been suggested to contribute to hypertensive conditions, at least in part, through the CNS [7]. However, future investigations are necessary to determine if exacerbated SFO insulin receptor signaling could be a culprit in cardiovascular disease pathogenesis, as well as delineation of the downstream networks/mechanisms through which SFO insulin receptors influence cardiovascular physiology.

### 3.2. OVLT

The OVLT is a tiny sensory CVO located at the rostral end of the third ventricle within the hypothalamic preoptic region [39]. Either in concert with the SFO or through independent mechanisms, the OVLT is also well recognized for its regulatory role in cardiovascular/autonomic, body fluid, and thirst physiology. For example, neurons within the OVLT sense extracellular [Na+] fluctuations in a dose-dependent manner, and OVLT-targeted NaCl treatment increases sympathetic nerve activity, blood pressure and heart rate [40,41]. These salt-sensitive OVLT neurons are also Ang-II type 1a receptor (AT1aR)-expressing glutamatergic neurons, suggested collectively to integrate cardiovascular, fluid, and sodium signals to influence blood pressure, through an OVLT-PVN pathway [41]. In line with this, alterations in the OVLT are also associated with several forms of neurogenic hypertension, including Ang-II, hypertonic salt-sensitive, and deoxycorticosterone acetate-salt-dependent hypertension [41,42,43].

When insulin is administrated peripherally or into the third ventricle, sympathetic nerve activity increases. This suggests a possible role for the OVLT in insulin-mediated sympathoexcitatory actions, as the OVLT is located around the third ventricle, and thus, could be an immediate target for central insulin. In support of this, previous work utilized peripheral administration of insulin under euglycemic conditions following surgical removal of the OVLT and surrounding areas (more specifically anteroventricular third ventricle regions including the OVLT, preoptic anterior hypothalamic nucleus and medial preoptic nucleus) [44]. Insulin failed to elicit increases in sympathetic nerve activity when the OVLT and surrounding areas were removed [44]. Although indirect, as the surgical removal included a wide range of brain regions surrounding the OVLT, and the fibers originating from the SFO to hypothalamic neuroendocrine and autonomic regions travel through this area, these findings support the anterior part of the hypothalamus as necessary for sympathoexcitation induced by insulin [44]. Following this investigation, another study to distinguish a role between the SFO and the OVLT was performed [45]. In anesthetized rats, the either the anteroventral third ventricle (which includes the OVLT) or SFO was surgically removed, and insulin-dependent lumbar sympathetic nervous activity was measured. Interestingly, only OVLT lesioning resulted in an attenuation of insulin-induced sympathoexcitation, suggesting that insulin-dependent sympathoexcitation is mediated, at least in part, by the OVLT, but not by the SFO [45]. While these findings point to a role for the OVLT in the regulation of the sympathetic nervous system by insulin, direct and specific evidence for the OVLT is still lacking. Similarly, despite this evidence, the involvement of the OVLT in insulin-associated cardiovascular regulation remains unexplored.

### 3.3. AP

In the brainstem, the AP is a sensory CVO located adjacent to the fourth ventricle that establishes direct synaptic communications with the nucleus tractus solitary (NTS) and dorsal motor nucleus (DMN) [46]. This AP-NTS-DMN complex is tightly associated both anatomically and functionally and is collectively considered as the dorsal vagal complex (DVC). Similar to the other sensory CVOs, the AP possesses the molecular and cellular machinery to sense circulating hormones, cytokines, nutrients, and peptides [47], and initiates neural cascades to regulate sympathetic nerve activity, blood pressure, heart rate, and baroreflex sensitivity, predominantly via the DVC [48,49]. For example, surgical removal of the AP blunts increases in cardiac sympathetic nerve activity in an animal model of heart failure [48], and additional studies indicate a key role for the AP in renovascular hypertension [49]. Therefore, alterations in the AP are directly implicated in the various cardiovascular disorders.

**Figure 2:**
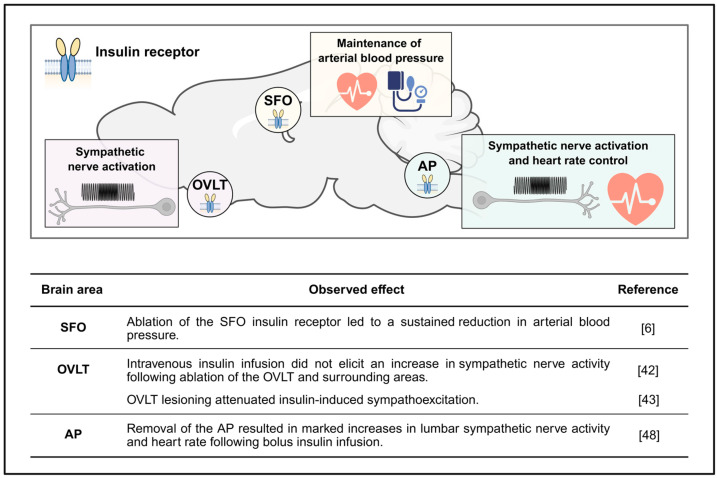
Schematic illustration and table summarizing the potential role for insulin receptors in the sensory CVOs in cardiovascular regulation. SFO, subfornical organ; OVLT, organum vasculosum lamina terminalis; AP, area postrema. Image was created with Biorender.com; accessed on 19 July 2025 [6,42,43,48].

A single study examining AP insulin action in relation to cardiovascular regulation has been reported [50]. Interestingly, removal of the AP resulted in exaggerated increases in lumbar sympathetic nerve activity and heart rate following bolus infusion of insulin. However, this effect disappeared when circulating glucose was balanced to euglycemic conditions, suggesting that the responses were related to hypoglycemia rather than removal of insulin signaling in the AP [50]. While findings are limited within the AP, a series of investigations to uncover NTS-specific insulin action in cardiovascular control have also been performed. Direct administration of insulin into the NTS lowers blood pressure and heart rate in anesthetized animals; this is mediated via the phosphoinositide 3-kinase-Akt pathway in concert with either nitric oxide or Wnt signaling [51,52]. However, this system is impaired in spontaneous as well as fructose-induced hypertensive rats [52,53], indicating a role for NTS insulin signaling in normal cardiovascular regulation as well as in the development of hypertension. Thus, similar to the existing data related to metabolic control, given the close anatomical relationship between the AP and NTS, the possibility exists that the AP may contribute to cardiovascular regulation through direct actions and/or by serving as the gateway for circulating insulin to access the NTS. (Figure 2)

## 4. Sensory CVOs Insulin Receptors in Metabolic Regulation

### 4.1. SFO

The SFO contains a wide array of metabolic receptors, including leptin, ghrelin, insulin, adiponectin, amylin, endocannabinoid, and Ang-II [32]. Moreover, as mentioned above, anatomically, the SFO establishes synaptic connections with numerous hypothalamic nuclei implicated in neuroendocrine and autonomic regulation [35]. In line with this molecular and anatomical profile of the SFO, emerging findings point to a unique role for the SFO in whole-body metabolism. For example, a role for SFO angiotensinergic signaling in leptin-induced weight loss and brown adipose tissue thermogenesis has been shown [54]. In addition, cellular stress mechanisms in the SFO have been recently shown to contribute to hepatic steatosis; a common comorbidity of obesity [55,56,57].

Based on the emerging evidence pointing to a metabolic regulatory role for the SFO, a recent report investigated the functional role of SFO insulin receptors in metabolic regulation [6]. Importantly, multiple independent investigations have confirmed dense expression of insulin receptors in the SFO [30,31,32]. Interestingly, selective viral removal of insulin receptors in the SFO resulted in enhanced body weight gain and adiposity in male mice fed a standard chow diet without influencing food intake [6]. In addition, ablation of SFO insulin receptors was also associated with the development of hepatic steatosis; findings that are similar to global neuronal knockout of insulin receptors [58]. These in vivo findings build upon previous electrophysiological approaches demonstrating a direct effect of insulin on a large majority of SFO neurons [30]. Together, the SFO has robust insulin receptor expression, SFO neurons are activated or deactivated in response to insulin, and SFO insulin receptors appear to be involved in body weight regulation along with adipose and liver metabolism. However, the detailed underlying molecular and cellular mechanisms involved have yet to be uncovered.

### 4.2. OVLT

Similar to the SFO, the OVLT has bi-directional synaptic outputs to CNS sites known to be important in energy homeostasis [41,59], and surgical dissection of neural communication in areas surrounding the OVLT and hypothalamic preoptic region attenuates body weight gain under standard diet conditions, independent of food intake, although the underlying mechanisms for this have not yet been elucidated [60]. Similarly, short-term chemical ablation of neural connections around the OVLT in rats, by direct administration of colchicine, decreases daily food intake and body weight gain [61].

Although the OVLT possesses the molecular machinery to sense insulin [6], investigations examining a direct effect of OVLT insulin signaling in metabolic physiology are nonexistent to date. However, corollary evidence has recognized a metabolic role for relaxin-3, a hormone belonging to the insulin superfamily with structural similarity to insulin [62]. Both peripheral and central administration of relaxin-3 produces OVLT neural activation and increases water and food intake [63]. Although indirect, these investigations point to a potential role for the OVLT in mediating relaxin-3-dependent metabolic regulation, and it is possible that insulin may mimic OVLT relaxin-3 actions. Moreover, insulin has been suggested to be associated with the anticipatory regulation of feeding, and investigations examining food anticipatory behavior using scheduled food restriction [64] or nursing rabbit pups [65] have recognized the involvement of the OVLT in such behavior. For example, neural activation accompanied by enhanced locomotor activity occurs prior to scheduled nursing time in rabbit pups, and mapping CNS sites with c-Fos, an immediate neuronal activity marker, pointed to neural activity in the OVLT as a key nucleus involved [65]. Therefore, insulin-associated anticipatory appetite behavior may occur through OVLT mechanisms. Nevertheless, the role of OVLT insulin signaling in metabolic regulation remains unknown and warrants future investigation.

### 4.3. AP

The DVC is well-recognized as an extra-hypothalamic brain center involved in the control of metabolism including glucose homeostasis [34], adiposity [66], appetite, body weight regulation [67], and brain-gut communication [68]. Historical and more recent evidence points to the AP as a CVO through which insulin influences metabolic-associated physiological responses. The AP is a core nucleus to induce vomiting in dogs and humans [69,70] and insulin excites AP neurons in a dose-dependent manner to induce emesis [69]. However, this insulin-dependent emesis disappears when the AP is removed, clearly indicating a role for AP insulin signaling in the emetic reflex [69]. In addition to emesis, AP insulin signaling is also associated with nutritional infertility. Body fuel availability is an important factor for reproductive behavior, and a decrease in fuel levels causes nutritional infertility. Hyperinsulinemia, insulin-dependent hypoglycemic stress, or food deprivation suppress reproductive behavior [71]. Importantly, this nutritional infertility is abolished when the AP is surgically removed [72]. Collectively, these findings highlight the AP as a key site involved in insulin-associated emesis and reproduction. However, examination of other metabolic roles (e.g., body weight, food intake, adipose, etc.) for insulin receptors in the AP have yet to be conducted.

**Figure 3:**
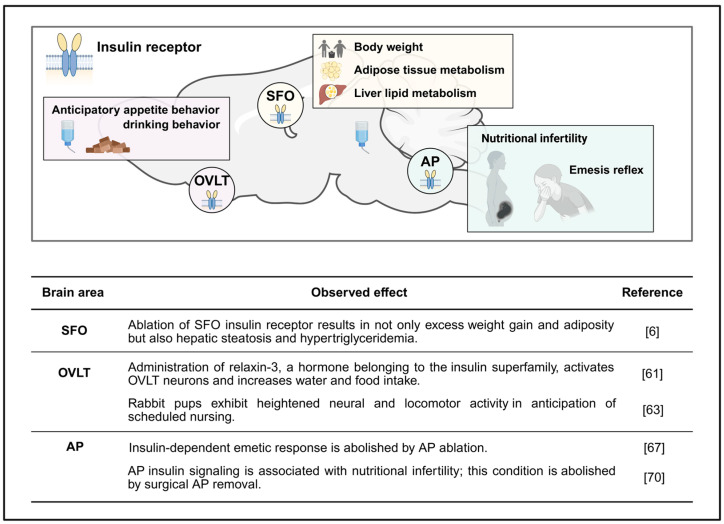
Schematic illustration and table summarizing the potential role for insulin receptors in the sensory CVOs in metabolism regulation. SFO, subfornical organ; OVLT, organum vasculosum lamina terminalis; AP, area postrema. Image was created with Biorender.com; accessed on 19 July 2025 [6,61,63,67,70].

It is important to note that the NTS and DMN are also implicated in insulin-mediated metabolism regulation. For example, NTS insulin receptors regulate whole-body glucose homeostasis and diet-specific appetite behavior [34,67], while insulin receptors in the DMN have been suggested to be involved in brain-gut communication [68]. Insulin resistance occurs in the DMN in diabetic conditions that are associated with gastrointestinal pathophysiology [68]. Given that the AP is anatomically adjacent to NTS and DMN, the AP may be the gate for circulating insulin to access these nuclei (Figure 3).

## 5. Summary and Perspectives

Insulin is a well-characterized cardiometabolic hormone due to its ability to modulate a diverse array of metabolic and cardiovascular parameters. Therefore, alterations in insulin signaling, particularly within the CNS, may be a key candidate that connects cardiovascular and metabolic disorders. However, our understanding of the brain site(s) involved in insulin action still remains unclear. The sensory CVOs are uniquely situated to initiate central insulin signaling to downstream autonomic and neuroendocrine nuclei, thus regulating cardiometabolic function. However, given the emerging, and somewhat limited investigations of insulin in the CVOs, it is important to consider further mechanistic insight into insulin-mediated cardiometabolic influences. For example, what are downstream CVO insulin-related signaling pathways involved? Insulin signaling is a complex process involving both the phosphoinositide 3 kinase (PI3K)/AKT and mitogen-activated protein kinase (MAPK) pathways [73,74]. Moreover, how might other cellular signaling pathways interact with CVO insulin action? Current evidence indicates that a variety of other hormones can influence insulin action. In brief, for example, activation of the renin-angiotensin system and hyperinsulinemia typically occur in parallel during hypertension, and blockade of the angiotensin-II type 1a receptor attenuates hyperinsulinemia-dependent hypertension [75]. Moreover, there is well documented evidence of inflammatory mediators impacting brain insulin signaling [25,76], which is intriguing given the documented role of inflammation in cardiometabolic conditions [77,78] and further the access of the CVOs to circulating inflammatory cytokines. Finally, it is important to consider the CVO cell type in which insulin signaling is occurring (neurons, astrocytes, microglia, endothelial cells, etc.) and additionally the downstream gene changes within specific cell types that may be influencing peripheral physiological processes.
